# The Predictors of Clinical Competence among Hospital Nurses: A Cross-Sectional Study in Hamadan, West Iran

**DOI:** 10.4314/ejhs.v33i1.5

**Published:** 2023-01

**Authors:** Keivan Babaei, Efat Sadeghian, Masoud Khodaveisi

**Affiliations:** 1 Ph.D Students in Nursing, School of Nursing and Midwifery, Hamadan University of Medical Sciences, Hamadan, Iran; 2 Chronic Diseases (Home Care) Research Center, Department of Nursing, Hamadan University of Medical Sciences, Hamadan, Iran; 3 Chronic Diseases (Home Care) Research Center, Department of Community Health Nursing, Hamadan University of Medical Sciences, Hamadan, Iran

**Keywords:** Clinical competence, Nurse, Care quality, Iran

## Abstract

**Background:**

Nurses' clinical competence (CC) is critical in providing high-quality and safe nursing care. Assessment of nurses' CC and its predictors is a key step to improve their CC and the quality of their services. The aim of this study was to determine the predictors of CC among hospital nurses in Iran.

**Methods:**

This analytical cross-sectional study was conducted from September 2020 to May 2021. Participants were purposively selected from four university hospitals in Hamadan, west of Iran. A demographic questionnaire and the 73-item Nurse Competence Scale were used for data collection. A total of 300 questionnaires were distributed and 270 questionnaires (response rate: 90%) were completed and returned to the researcher. Data were analyzed using the SPSS software (v. 16.0) and the one-way analysis of variance, the independent-sample t, the Mann-Whitney U, and the Kruskal-Wallis tests, the Pearson and the Spearman correlation analyses, and the linear regression analysis.

**Results:**

The mean score of CC was 40.28±8.6 (in the possible range of 0–100) and the highest and the lowest dimensional mean scores were for the situation management (56.13±11) and the ensuring quality (25.3±8.1) dimensions, respectively. The mean score of CC had significant relationship with age, work experience, and ward of working and these variables significantly predicted 77% of the variance of CC (adjusted R = 0.778, P<0.05).

**Conclusions:**

According to the results of this study, age, work experience and ward of working weresignificant predictors of CC in hospital nurses. Nursing managers should employ strategies such as reducing nurses' workload, improving their employment status, and providing them with quality in-service education in order to improve their CC and the quality of their services.

## Introduction

Nurses, as the largest group of healthcare providers, have significant role in delivering quality care services and achieving the goals of healthcare delivery (1). Their roles and responsibilities in healthcare systems include clinical leadership and management, safety improvement, and evidence-based practice, and hence, they need to have adequate medical, technical, and communication skills (2). Quality care delivery by nurses is associated with patient satisfaction and better quality improvement indicators (3), while low quality care delivery can lead to different problems such as patient fall, sepsis, pneumonia, urinary tract infection, pressure ulcer, and re-hospitalization (4).

The goal of health care is to provide safe, high-quality care, and this places nurses' competence in focus. Clinical competence (CC) is a fundamental component of nursing care and plays an important role in the quality of services provided by nurses (5). Derived from the Middle France and Latin, competency means right pertinence, adequate qualification, and adequate ability or capacity to do something (6). Competence in nursing practice is complex and therefore difficult to assess and there is no consensus regarding the definition of competence. There are different definitions for the concept of CC, each referring to certain aspects or dimensions of CC. A study reported that CC consists of seven skills, namely helping, teaching-coaching, diagnostic functions, situation management, therapeutic interventions, ensuring quality, and work role (7). Another study found that the dimensions of CC were critical thinking, clinical care, leadership, interpersonal relationships, ethical practice, professional development, and teaching-coaching (8). However, it has been indicated that competence in nursing is a dynamic process rather than the sum of individual competencies (7–8).

CC is associated with many different outcomes. Nurses' adequate CC has significant positive relationship with patient satisfaction (9), patient safety, nursing profession reputation (10–11), quality of working life (12), self-confidence, safe nursing practice, holistic care delivery, quality multidisciplinary relationships, effective patient need assessment, effective patient management, and emotional support for patients and their families (13). Moreover, CC has negative relationship with occupational stress, so that nurses with lower CC experience higher occupational stress (11).

Previous studies reported various findings regarding the level of nurses' CC and its dimensions in Iran (7, 14–15). CC is a multifactorial concept with many different contributing factors. The results of a systematic review and meta-analysis indicated that some individual and occupational factors such as age, educational level, work experience, income, employment status, and job satisfaction can significantly affect CC of nurses (16). However, there are limited data about the contributing factors of CC among hospital nurses in Iran, and conducting more studies in this regard has been suggested to narrow this gap (7). Therefore, this study aimed to determine the level of CC and its predictors among hospital nurses in Iran.

## Methods

The strengthening the reporting of observational studies in epidemiology (STROBE) checklist was used to report important aspects of this study.

**Study design**: This analytical cross-sectional study was conducted from September 2020 to May 2021.

**Setting:** Study setting was the emergency departments, internal medicine and surgical wards and the intensive care units (ICU_s_) of the 200-bed Shahid Beheshti, the 600-bed Besat, the 200-bed Sina, and the 300-bed Farshchian hospitals, Hamadan, west of Iran. It has been previously indicated that the different levels of clinical competency of nurses in these wards may be considered representative of clinical competency of hospital nurses (7, 11–12).

**Participants**: Study population comprised all nurses working in these wards (around 1000 nurses). Nurses were selected by stratified sampling method based on their ward of working. Then, systematic random sampling approach was used inside each wards for recruiting the nurses in the study sample.

Eligible participants were purposively selected based on these criteria: bachelor's or master's degree, employment as hospital nurse in morning or rotating shift, hospital work experience more than one year, and work in emergency or internal medicine and surgical wards or intensive care unit. Incomplete answer to the questioners was an exclusion criterion.

**Data sources/ measurement**: Data collection instruments were a demographic questionnaire and the Nurse Competence Scale. The demographic questionnaire had items on age, gender, work experience, affiliated ward, educational level, and work shift. The Nurse Competence Scale has 73 items in seven dimensions, namely helping role (seven items), teaching-coaching (sixteen items), diagnostic functions (seven items), situation management (eight items), therapeutic interventions (ten items), ensuring quality (six items), and work role (nineteen items). Items are scored on a 0–100 visual analogue scale on which zero stands for “Very low competence” and 100 stands for “Very high competence”. The possible total scores of the scale and its dimensions are 0–100 and are interpreted as low CC (scores 0–25), moderate CC (scores 25.1–50), high CC (scores 50.1–75), and excellent CC (scores 75.1–100). Meretoja et al. developed and psychometrically evaluated this scale using the data obtained from 498 nurses (17). A study in Iran also confirmed the validity and reliability of the Persian version of this scale with Cronbach's alpha values of 0.75–0.89 for its dimensions (18). In this study the reliability of the questionnaire was determined using test-retest and Cronbach alpha coefficient (α=0.79). A total of 300 questionnaires were distributed and 270 questionnaires were completed and returned to the researcher (response rate: 90%). Participants personally completed the study instruments at their workplace.

**Sample size calculation**: The estimation of sample size was based on a presumed effect size of 0.3, a statistical power of 95%, and a type I error of 5% using G*Power software, version 3.1.3 with the formula for calculation of sample of correlational studies. The overall proper sample size was found to be 300 participants.

**Ethical considerations**: The study followed the ethical principals in accordance with the Declaration of Helsinki. The Institutional Review Board and the Ethics Committee of Hamadan University of Medical Sciences approved this study (codes: 9905072878 and IR.UMSHA.REC.1399.249, respectively). Necessary permissions for sampling and data collection were obtained from the deputy of research and technology of Hamadan University of Medical Sciences, Hamadan, Iran, and provided to the authorities of the study setting. Before data collection, participants were informed about the study aims and methods and confidentiality of the data and their informed consent was secured. Nurses' participation was voluntary and they could end their participation at any time without explanation. All study instruments were anonymous.

**Statistical analysis**: Statistical Package for the Social Sciences (SPSS) software package (version 16.0, SPSS Inc., Chicago, IL, USA) was used for data analysis. Normality of the data was evaluated using the Kolmogorov-Smirnov test. Data were described using the measures of descriptive statistics, namely mean, standard deviation, and frequency. The relationship of the mean scores of CC and its dimensions with other variables were assessed using the independent-sample *t* or the non-parametric Mann-Whitney *U* test, the analysis of variance or the non-parametric Kruskal-Wallis test, and the Pearson or the nonparametric Spearman correlation analysis. The predictors of CC were also determined using linear regression. Variables with significant relationship with CC at a significance level of less than 0.1 were entered into the regression model. The level of significance was set at less than 0.05. No missing items were accepted in the calculation of CC domains and total scores.

## Results

A total of 300 hospital nurses were selected to answer the study instruments. Thirty participants provided incomplete answers to the instruments and were excluded and final analysis was conducted on the data collected from 270 participants. The mean of participants' age was 33 years in the range of 24–52. Most participants were female (63%; n=170), had bachelor's degree (91%; n=247), worked rotating shifts (83%; n=225), and had a work experience of less than three years (68%; n = 185). Moreover, 38% of participants (n=105) worked in emergency departments, 35% of them (n=96) worked in internal--medicine and surgical wards, and 27% of them (n=69) worked in intensive care unit (Table 1). The mean score of participants' CC was 40.28±8.6 in total, 31.34±10 in the helping role dimension, 40.11±11 in the teaching-coaching dimension, 42.58±13 in the diagnostic functions dimension, 56.13±11 in the situation management dimension, 40.44±9 in the therapeutic interventions dimension, 25.3±8 in the ensuring quality dimension, and 39.62±8 in the work role dimension (Table 2).

**Table 1:** Participants' characteristics and their relationships with CC and its dimensions

CC dimensions		Helping role	Teaching-coaching	Diagnostic functions	Situation management	Therapeutic interventions	Ensuring quality	Work role	Total
Characteristics									
Gender	Male	29.21±9	37.15±10	40.25±13	56.16±12	39.92±8.4	23.91±7.5	39.66±9.4	38.89±8.3
	Female	34.11±10	43.12±11	45.3±12	57.32±10	41.75±10.2	27.11±8.5	39.69±8.3	41.72±8.9
	P value	0.011¶	0.028¶	¶0.007	¶ 0.555	Φ 0.241	¶0.086	Φ0,748	Φ 0.099

Level of	Bachelor's	30.94±10	40/5±12	42.21±13	57.47±11	40.21±10.1	22.22±6.6	39.17±9	40.31±9.3
education	Master's	33.62±8	40.86 ±16	43.73±12	58.23±9	40.89±7.6	33.45±5	39.44±5.4	40.62±7.1
	P value	0.174¶	¶0.979	¶0.642	¶0.082	Φ0,792	¶<0.001	Φ 0.422	* 0.767

Ward	ED	22.78±5	31.22±5	29.81±8	39.15±4	32.11±6.3	21.66±8	31.72±4.7	32.84±4.4
	MSW	32.15±7.5	38.32±7	42.27±63	44.13±8.5	41.88±9.7	25.56±7	38.29±4.7	38.88±6.3
	ICU	39.65±4	52.11±9	56.16±9	66.42±6.8	48.11±2	29.13±8	48.11±6.4	49.48±3.8
	P value	⦵<0.001	⦵<0.001	⦵<0.001	⦵<0.001	⋔<0.001	⦵<0.001	⋔<0.001	⋔<0.001

Work experience	Little (≤5 years	29.11±10	39.33±11	39.72±12	54.41±10	38.94±9.9	25.15±9	36.33±6.5	38.11±8
	Great (>5 years)	35.6±8.5	43.6±12	49.17±12.8	62.58±11	43.66±7.4	25.38±7	45.86±9	44.48±8.3
	P value	¶<0.001	¶0.027	¶<0.001	¶<0.001	Φ 0.019	¶0.837	Φ<0.001	Φ< 0.001

Age	r	0.183	0.157	0.235	0.321	0.107	-0.032	0.396	0.256
(Years)	P value	0.069*	0.119*	0.018*	O.001*	0.289^	0.749*	<0.001^	0.01^

¶ The results of the Mann-Whitney *U* test

Φ The results of the independent-sample *t* test

⦵ The results of the Kruskal-Wallis test

⋔ The results of the one-way analysis of variance

* The results of the Spearman correlation analysis

^ The results of the Pearson correlation analysis, ED: Emergency department , MSW: medical surgical ward, ICU: intensive care unit

**Table 2:** The mean scores of CC and its dimensions

CC dimensions	Mean (SD)	Range
Total	40.28±8.6	27.3–57.1
Helping role	31.34±10	17.14–70
Teaching-coaching	40.11±11	6.25–65.6
Diagnostic functions	42.58±13	22.8–64.2
Situation management	56.13±11	35–77.5
Therapeutic interventions	40.44±9	26–80
Ensuring quality	25.3±8	13.3–43.3
Work role	39.62±8	26.3–58.4

Accordingly, the highest and the lowest dimensional mean scores were for the situation management and the ensuring quality dimensions, respectively. The highest scored item in the situation management dimension was the “Able to recognize situations posing a threat to life early” item and the lowest scored item in the ensuring quality dimension was the “Utilizing nursing research findings in relationships with patients” item.

The mean scores of the helping role, teaching-coaching, and diagnostic functions dimensions of CC among female participants were significantly more than male participants (P<0.05), while there were no significant differences between male and female participants respecting the mean scores of total CC and its other dimensions (P>0.05). Moreover, there were no significant differences between bachelor's and master's degree nurses respecting the mean scores of CC and its dimensions (P>0.05), except for the mean score of the ensuring quality dimension which was significantly greater among master's degree nurses (P<0.001).

There were no significant differences between nurses who worked morning shift and those who worked rotating shifts respecting the mean scores of CC and all its dimensions (P>0.05). The differences among nurses in emergency ward, internal medicine ward, and intensive care unit respecting the mean scores of CC and all its dimensions were also significant so that these mean scores in nurses in intensive care unit were significantly greater than other nurses and in nurses in internal medicine ward were significantly greater than nurses in the emergency ward (P<0.05). Moreover, compared with nurses with lower work experience, nurses with higher work experience obtained significantly higher scores for CC and its dimensions (P<005), except for the ensuring quality dimension (P=0.837). Participants' age also had significant positive correlation with the mean scores of CC and its situation management, diagnostic functions, and work role dimensions (P<0.05) (Table 1). Variables with significant relationship with the total mean score of CC at a level of less than 0.1 were entered into the linear regression model. Results revealed that age, affiliated ward, and work experience significantly predicted 77% of the variance of CC (adjusted R=0.778) (Table 3).

**Table 3:** The results of the linear regression analysis to determine the predictors of CC.

Variables	Results	B	Std. Error	Beta	*t*	P value
**Age (Years)**		1.62	1.12	0.129	1.44	0.034
**Work experience**	Little (≤5 years)	—	—	—	—	—
	Great (>5 years)	5.79	1.2	0.427	4.8	<0.001
**Ward**	Emergency department	—	—	—	—	—
	Internal medicine and surgical ward	3.58	0.74	0.264	4.8	<0.001
	Intensive care unit	12.06	0.72	0.903	16.5	<0.001
**R square= 0.787**	**adjusted R square = 0.778**					

## Discussion

The results of this study showed that CC of nurses was moderate and age, work experience, and ward of working was significant predictors of CC in hospital nurses. Similarly, two studies reported that nurses in intensive care units and emergency departments had moderate CC and higher CC score than nurses who worked in other wards (15). The results of a study by Mirlashari et al. showed that there was a direct statistically significant relationship between marital status, employment status, level of interest in working and the CC of nurses (19). However, another study showed that there is no statistically significant difference between the CC score of nurses and their gender, age, academic degree, and work experience (20). It has been shown that limited in-service training, shortage of equipment, and nurses' limited sense of responsibility (20–21) can reduce nurses' CC. In some countries critical care nurses need to pass CC courses and have CC certificate as a prerequisite for employment. CC certificate is a major factor contributing to CC development in these countries (22–23). Moreover, factors such as low income (24), income-related job dissatisfaction (16, 25), and job burnout can reduce nurses' CC (26–29).

Our findings also indicated age and work experience as significant predictors of CC, so that nurses with higher age and work experience had greater CC. Similarly, a study on nurses in Iran reported that age and work experience had significant effects on CC (21). Several other studies in different countries also reported the significant correlation of age and CC (30–32). Older nurses usually have greater CC due to their greater work experience. Great work experience improves nurses' self-confidence and helps them predict the course of a clinical situation and make sound decisions based on similar previous experiences or situations (33–34).

We also found that the mean scores of the situation management and the work role dimensions of CC among older nurses were significantly higher than younger nurses. The situation management dimension relates to the identification of life-threatening conditions, appropriate actions in such situations, education for healthcare providers to improve their control over such situations, and improvement of healthcare providers' collaboration to manage rapid changes. The work role dimension also refers to the awareness of limitations in personal resources, self-care in physically and mentally stressful work conditions, development of personal knowledge to improve patient care, delegation of authority, and appropriate and committed actions in relation to the limited financial resources of the organization (7). Therefore, experienced nurses should be involved in providing education to young nurses.

Study findings also revealed that nurses' educational level had no significant effects on their CC, which is in agreement with the findings of two former studies (35–36). Contrarily, nurses with higher educational level in two other studies had greater CC (37–38). In a study in Sweden it has been shown that emergency nurses' grater clinical experience and advanced levels of education were positively associated with their competency (39).

Another finding of the present study was that the mean score of the ensuring quality dimension of master's degree nurses was significantly higher than their bachelor's degree counterparts. The ensuring quality dimension relates to nurses' perception of patient satisfaction, use of research findings in care delivery, and identification of care-related aspects which need further assessment and development (7). This contradiction may be due to the differences among these studies respecting their participants' characteristics such as age and work experience. CC consists of knowledge, skills, experiences, and attitudes (40) and hence, pure development of theoretical knowledge through graduate courses, like some master's courses in Iran, may not be effective in its significant improvement. The higher score of this dimension among master's degree nurses in Iran may be due to their greater knowledge of nursing research, biostatistics, health information technology, nursing ethics, legal issues, and professional communication. Moreover, master's courses in Iran integrate workshops on evidence-based practice, communication skills, and interviewing, and hence, can improve nurses' competence in quality care delivery.

Gender had no significant effects on CC in the present study, which is in line with the findings of a previous study in Iran (21). However, the mean scores of the helping role, teaching-coaching, and diagnostic functions dimensions of CC among female participants were significantly more than male participants. This finding may be due to female nurses' greater abilities in physical examination and history taking and their closer adherence to professional principles and human values.

Nurses' affiliated hospital ward was another predictor of CC in the present study so that nurses in intensive care unit had the greatest CC and nurses in the emergency ward had the lowest CC. This is in agreement with the findings of a previous study in Iran (20). The greater CC of critical care nurses in the present study may be due to the more critical conditions of patients in intensive care units which necessitate the employment of experienced and competent nurses in these units. Moreover, critical care nurses usually have better critical thinking skills than other nurses. Critical thinking consists of the ability to think about complex problems, diagnose potential and actual health problems, and establish effective communication with patients and healthcare providers.

In contradiction with our findings, a study in South Korea reported no significant relationship between CC and nurses' affiliated ward (37). This contradiction may be due to the difference between the studies with respect to their participants' affiliated wards so that 51.2% of nurses in the study in Korea worked in general hospital wards (38), while 35% of our participants worked in intensive care unit.

This study has some limitations that need to be addressed. First, the cross-sectional design of this study allows for a demonstration of association between certain factors and CC of Iranian nurses, but limits our ability to form firm conclusions regarding causality. Second, the result of this study may not be generalizable to other nurses in developing countries with different health professional, socio-cultural, economic, and political backgrounds and also nurses who work in different wards in hospitals. In conclusion, according to the results of this study it seems that Iranian hospital nurses have moderate CC and age, work experience, and ward of working was significant predictors of CC in hospital nurses.
